# A Physical Interaction between the Dopamine Transporter and DJ-1 Facilitates Increased Dopamine Reuptake

**DOI:** 10.1371/journal.pone.0136641

**Published:** 2015-08-25

**Authors:** Beryl Luk, Mohinuddin Mohammed, Fang Liu, Frank J. S. Lee

**Affiliations:** 1 Faculty of Health Sciences, Simon Fraser University, Burnaby, British Columbia, Canada; 2 Centre for Addiction and Mental Health, University of Toronto, Toronto, Ontario, Canada; Institute for Interdisciplinary Neuroscience, FRANCE

## Abstract

The regulation of the dopamine transporter (DAT) impacts extracellular dopamine levels after release from dopaminergic neurons. Furthermore, a variety of protein partners have been identified that can interact with and modulate DAT function. In this study we show that DJ-1 can potentially modulate DAT function. Co-expression of DAT and DJ-1 in HEK-293T cells leads to an increase in [^3^H] dopamine uptake that does not appear to be mediated by increased total DAT expression but rather through an increase in DAT cell surface localization. In addition, through a series of GST affinity purifications and co-immunoprecipitations, we provide evidence that the DAT can be found in a complex with DJ-1, which involve distinct regions within both DAT and DJ-1. Using *in vitro* binding experiments we also show that this complex can be formed in part by a direct interaction between DAT and DJ-1. Co-expression of a mini-gene that can disrupt the DAT/DJ-1 complex appears to block the increase in [^3^H] dopamine uptake by DJ-1. Mutations in DJ-1 have been linked to familial forms of Parkinson’s disease, yet the normal physiological function of DJ-1 remains unclear. Our study suggests that DJ-1 may also play a role in regulating dopamine levels by modifying DAT activity.

## Introduction

Dopaminergic neurotransmission is mediated by volume transmission that is largely extrasynaptic and is regulated by the levels of dopamine (DA) in the extracellular space [1–8]. One of the major mechanisms for regulating DA levels is through reuptake via the dopamine transporter (DAT). In addition to DA, the DAT also facilitates the reuptake of the neurotoxin 1-methy-4-phenylpyridinium (MPP+), which induces symptoms resembling Parkinson’s disease (PD) [9–12]. Furthermore, DA has been implicated in numerous disease states including schizophrenia, drug abuse and PD [13–16]. DAT regulation can be mediated by various means including activation of PKC [17–22], PKA [22–25], CaMKII [25–28] and tyrosine kinase [29–32]. In addition, N-linked glycosylation affects DAT maturation and localization to the plasma membrane [33,34]. DAT endocytosis has also been demonstrated to occur by ubiquitination mediated by the E3 ubiquitin ligase Nedd4-2 [35]. Interestingly, studies have identified several proteins that can physically couple to DAT and regulate DAT-mediated reuptake. These proteins include the dopamine D2 receptor [36,37], PICK1 [38], Hic-5 [39], syntaxin1A [40–42], GPR37 [43], piccolo [44], synaptogyrin-3 [45], CaMKII [28], G protein βγ subunits [46] and α-synuclein [47,48], to name a few. Notably, α-synuclein, in which mutations have been linked to familial forms of PD, binds to the DAT carboxyl terminus (CT) and modifies DAT uptake. In this report we have identified that another PD-linked gene, DJ-1/PARK7, has an effect on DAT function.

The human DJ-1 gene encodes a protein of 189 amino acids [28]. Although the main physiological role of DJ-1 remains unclear, several groups have implicated DJ-1 in oxidative stress, the ubiquitin-proteasome system, mitochondrial activity and the dopaminergic system (reviewed in [49] and [50]). Moreover, most mutations in the DJ-1 gene have been linked to autosomal recessive early-onset PD [51–53]. Given that pathological DJ-1 mutants are loss-of-function mutations, several knockout mice have been generated [54–57]. These mice appear to exhibit altered DA reuptake [54,56] and a decrease in D2 autoreceptor function [54,57], two key elements in regulating extracellular DA levels. Consequently, the brains of mice lacking DJ-1 exhibit increased DA tissue content. Furthermore, DJ-1-null mutant mice exhibit enhanced sensitivity to 1-methyl-4-phenyl-1,2,3,6-tetrahydropyridine (MPTP) resulting in increased striatal denervation by DA neurons [55,56]. This increased susceptibility to MPTP in DJ-1 null mice was ameliorated by viral-mediated expression of DJ-1 [55]. Thus, DJ-1 may be a key protein that is involved in regulating DA levels by differentially regulating dopaminergic proteins, including the DAT and perhaps modifying the susceptibility of dopaminergic neurons to neurotoxic insults. Furthermore, loss-of-function mutations in the DAT gene have been shown to be associated with autosomal recessive infantile parkinsonism dystonia [58] and with adult parkinsonism [59]. Taken together, this implicates the importance of DAT regulation. Therefore, in this study we explored the possibility that DJ-1 can directly bind to the DAT and regulate transporter activity.

## Materials and Methods

### GST fusion proteins and mini-genes

DJ-1 cDNA was kindly provided by Dr Jin Xu [60]. DAT and DJ-1 cDNA fragments were amplified by PCR from full-length cDNA clones. All 5’ and 3’ oligonucleotides incorporated BamHI and EcoRI sites, respectively, to facilitate sub-cloning into pcDNA3 or pGEX4T-3 [15, 39]. Initiation methionine residues and stop codons were also incorporated where appropriate. GST fusion proteins were prepared from bacterial lysates as described by the manufacturer (GE Life Sciences, Pittsburgh, PA). To confirm appropriate splice fusion and the absence of spurious PCR generated nucleotide errors, all constructs were re-sequenced.

### Fusion protein generation and purification

For protein expression in bacteria, BL21 *Escherichia coli* were transformed with either recombinant pGEX-4T3 or pET-28a. Briefly, bacterial cultures were grown at 37°C for two hours followed by induction with 500 μM IPTG and then returned to 37°C for 2 hours. Cultures were resuspended in ice-cold phosphate buffered saline (PBS) (137 mM NaCl, 2.7 mM KCl, 10 mM Na_2_HPO_4_, 1.8 mM KH_2_PO_4_) with 1% Triton X-100, allowed to mix for 10 minutes at 4°C before being sonicated for 60 seconds. Samples were centrifuged to pellet the insoluble fraction. For GST fusion protein purification, 50 μl of 50% glutathione-agarose beads (Sigma-Aldrich, Oakville, ON, Canada) in PBS was added to 1 ml of bacterial lysates, mixed overnight at 4°C, and washed three times for 5 minutes with cold PBS at room temperature. The protein was eluted with elution buffer (20 mM reduced L-glutathione, 100 mM Tris (pH 8), 120 mM NaCl). His-tagged protein was purified according to the manufacturer’s instructions (Sigma-Aldrich). In brief, 50 μl HIS-Select nickel affinity gel was equilibrated with equilibration buffer (150 mM, NaCl, 50 mM NaH_2_PO_4_; pH 8.0) and then added to 1 ml of bacterial lysate, mixed at room temperature for 2 hours and washed three times with wash buffer (150 mM NaCl, 50 mM NaH_2_PO_4_; pH 8.0). The His-tagged protein was eluted with elution buffer (50 mM NaH_2_PO_4_, 300 mM NaCl, 250 mM imidazole; pH 8.0). The Quick Start Bradford kit from Bio-Rad Laboratories (Mississauga, ON, Canada) was used to determine protein concentrations by reading absorbance values at 595 nm on a Victor Plate Reader (PerkinElmer, Woodbridge, ON, Canada).

### Affinity purification assay, co-immunoprecipitation and Western blotting

Transfected HEK-293T cells were homogenized in modified RIPA buffer (50 mM Tris-HCl (pH 7.5), 150 mM NaCl, 1% NP-40, 0.5% sodium deoxycholate, 2 mM EDTA, 1 mM sodium orthovanadate, 0.1% Triton X-100) and Complete protease inhibitor cocktail (Roche, Indianapolis, IN) and centrifuged at 16,100 x g for 15 minutes at 4°C. 500–750 μg of protein extracts were incubated with 50 μg of GST fusion protein overnight at 4°C. Next day, 25–50 μl of 50% glutathione-agarose beads (in PBS) was added and incubated at room temperature for 2 hours. Beads were washed three times with 500 μl PBS containing 0.05–0.1% Triton X-100 and boiled for 10 minutes in SDS sample buffer. Samples were resolved by SDS-PAGE followed by Western blotting. For co-immunoprecipitation experiments, 25 μl Pierce Protein A/G agarose beads (Thermo Fisher Scientific, Ottawa, ON, Canada) were incubated in the presence of 2 μg of anti-DJ-1 (Enzo Life Sciences, Farmingdale, NY) or IgG (Jackson ImmunoResearch Laboratories, West Grove, PA) for 2 hour at 4°C, followed by the addition of 500–750 μg of HEK-293T cell lysates (homogenized in modified RIPA buffer) and mixed overnight at 4°C. Agarose beads were washed three times with PBS and boiled for 10 min in SDS sample buffer and subjected to SDS-PAGE. For experiments using rat striatum, tissue was homogenized in modified RIPA buffer with Complete protease inhibitor cocktail (Roche, Indianapolis, IN). A Kontes pestle (Thermo Fisher Scientific, Ottawa, ON, Canada) was used to homogenize the tissue with 50–100 up and down strokes. Tissue samples were placed on a rocker at 4°C for 1 hr and was subsequently centrifuged at 16,100 x g for 15 minutes at 4°C. Supernatant lysates were collected and 500–750 μg of total protein was used for co-immunoprecipitation experiments as previously described. Co-immunoprecipitation samples were subjected to electrophoresis on 10% gels (SDS–PAGE). Gels were transferred to polyvinylidenedifluoride (PVDF) membrane (Bio-Rad Laboratories, Mississauga, ON), blocked with 5% non-fat milk in TBS-T buffer (10 mM Tris-HCl, 150 mM NaCl and 0.1% Tween-20) for 1 hour at room temperature, washed three times and incubated with primary antibody, at appropriate dilutions in TBS-T, overnight at 4°C. Primary antibodies used in western blots were all purchased from Santa Cruz Biotechnology (Santa Cruz, CA) and used at a 1:200 dilution (DAT, sc-32258; DJ-1, sc-27004; α-tubulin, sc-8035). Following three TBS-T washes, the blot was incubated with appropriate secondary antibody (diluted 1:12,000 in TBS-T with 0.5% milk) for 1–1.5 hours at room temperature. The blots were visualized with SuperSignal West Dura Extended Duration Substrate (Thermo Fisher Scientific, Ottawa, ON) with Dyversity image analysis system and GeneSnap image acquisition software (Syngene, Frederick, MD).

### In vitro binding assays

For affinity purification using His-tagged protein as bait, 10 μg His-tagged protein and 0.5 μg GST fusion protein was incubated overnight at 4°C. 10 μl of equilibrated HIS-Select nickel affinity gel (Sigma-Aldrich) was added at room temperature for 2 hours. Beads were washed three times with cold PBS with 0.05% Triton X-100 (centrifuged at 4°C at 400 x g), boiled for 10 minutes in SDS sample buffer, resolved by SDS-PAGE followed by Western blotting with GST primary antibody (Cell Signalling Technology, Danvers, MA; 1:2,000 dilution) and goat anti-rabbit IgG secondary antibody (Jackson ImmunoResearch Laboratories).

### Cell culture and transfection of cDNA

A subtype of HEK-293 cells that express a plasmid containing the temperature sensitive mutant of SV-40 large T-antigen (HEK-293T) [61], were cultured in complete media, which consisted of DMEM (Life Technologies) supplemented with 10% FBS (Life technologies), passaging every 3–4 days. On the day prior to transfection, cells were seeded into 24 well plate (uptake assays, cell surface ELISAs) or 35 mm plates (live cell imaging) at 25–30% confluency. Cells were transfected using a calcium phosphate protocol. Briefly, on the day of transfection, media was changed to serum-free DMEM. Total DNA amounts used were 20 μg/plate (24 well plate) or 5 μg/plate (35 mm plate), whereby the proportion of DAT cDNA used was 1/5 to 1/8 of the total DNA used for the transfection, with remaining DNA consisting of either empty pcDNA3 plasmid (for control cells) or DJ-1 cDNA. The DNA/calcium precipitate mixture was added dropwise to plates and left on the cells for 4 hours upon which the media was changed to complete media. Cells were used in experiments 24–48 hours after the start of the transfection.

### [^3^H]DA Uptake Analysis

Measurement of DA uptake was performed on intact cells as previously described [36,47]. Briefly, HEK-293T cells were transfected with human DAT cDNA using calcium phosphate. Two days following transfection in 24-well plates (8.3 x 10^4^ cells seeded per well) medium was removed and wells were rinsed with 0.5 ml of uptake buffer (5 mM Tris, 7.5 mM HEPES, 120 mM NaCl, 5.4 mM KCl, 1.2 mM CaCl2, 1.2 mM MgSO4, 1 mM ascorbic acid, 5 mM glucose; pH 7.1). Cells were preincubated with 1 mM tropolone (Alfa Aesar, Ward Hill, MA) and 100 μM pargyline hydrochloride (Cayman Chemical Company, Ann Arbor, MI) for 5 minutes prior to the addition of 20–40 nM [^3^H] DA (PerkinElmer, Woodbridge, ON, Canada) and incubated for 10 minutes at room temperature in a total volume of 0.5 ml. Nonspecific [^3^H] DA (21.2–24.3 Ci/mmol) uptake was defined in the presence of 10 μM GBR 12909 dihydrochloride (Sigma-Aldrich). Wells were rinsed twice with 0.25 ml of uptake buffer and once with 0.5 ml uptake buffer, and cells were solubilized in 0.5 ml of 1% SDS and collected to measure incorporated radioactivity using a Beckman liquid scintillation counter (LS 6000SC). For saturation experiments, cells were pre-incubated in duplicate with increasing concentrations of nonradioactive dopamine (10^−9^ to 10^−4^ M) 5 min before the addition of 0.25 mL of 20 nM [^3^H] dopamine (final concentration) and incubated for 10 min at room temperature in a total volume of 0.5 ml. Non-specific [3H] dopamine uptake was defined in the presence of 10 uM GBR12909. For all experiments, direct assay comparisons between co-transfections and single transfections were conducted in parallel, using the same dilutions of drug, on the same batch of transfected cells.

### Cell surface ELISA

Cell-ELISA assays (colorimetric assays) were done essentially as previously described [36]. HEK-293T cells were fixed in 2% paraformaldehyde for 10 min. Some samples were permeabilized with 0.5% Triton X-100 in PBS. All samples were incubated in 5% skim milk in PBS-Tween (0.1%). Cells were incubated with DAT antibody against the extracellular loop (Santa Cruz Biotechnology, Santa Cruz, CA) under non-permeabilized (cell surface DAT pool) or permeabilized Conditions (total DAT pool). After incubation with corresponding HRP-conjugated secondary antibodies (Sigma), HRP substrate OPD (Sigma) was added and reaction was stopped with 3N HCl. The cell surface expression of DAT was presented as the ratio of colorimetric readings under non-permeabilized conditions to those under permeabilized conditions. Analysis was performed using at least 6 separate dishes in each group.

### Live cell fluorescence imaging of HEK-293T cells

Twenty four hours after transfection, HEK-293T cells plated on glass coverslips and transfected with CFP-DAT, DJ-1-YFP or the empty expression plasmid pcDNA3 were washed three times with Tyrode buffer (129 mM NaCl, 2.5 mM CaCl2, 5 mM KCl, 3 mM MgCl2, 30 mM glucose, 25 mM HEPES pH 7.4) then mounted into an imaging chamber. The cells were examined using an inverted epifluorescence microscope (Olympus IX81, Richmond Hill, ON, Canada), and images were captured by CoolSNAP HQ2 CCD camera (Photometrics, Tucson, AZ). Images were collected under 60X objective lens using Metamorph software (Molecular Devices, Sunnyvale, CA). For capturing CFP images, a 427/10 excitation filter and 472/30 emission filters mounted on separate filter wheels (Sutter Instruments, Novato, CA) were used. For capturing YFP images, a 504/12 excitation filter and 542/27 emission filters were used. In both cases a dual band dichroic mirror cube (440/520) was used.

### siRNA mediated knockdown of DJ-1

DJ-1 knockdown was mediated with the use of the TriFECTa RNAi Kit (Integrated DNA Technologies Inc., #HSC.RNAI.N007262.12). HEK-293T cells were transfected with either the control NC1 siRNA duplex (control) or DJ-1 siRNAs (Integrated DNA Technologies Inc., #HSC.RNAI.N007262.1, #HSC.RNAI.N007262.2, #HSC.RNAI.N007262.3; siDJ-1#1, siDJ-1#2 and siDJ-1#3, respectively). On the day of the transfection, media was replaced with fresh complete media. HEK-293T cells were transfected with 25 nM siRNA using jetPrime transfection reagent (Polyplus-transfection SA) according to manufacturer’s protocol. Cells were allowed to incubate with the transfection mix for 24 hours at which point the media was replaced with fresh complete media.

### APP+ uptake

4-(4-(dimethylamino)phenyl)-1-methylpyridinium (APP+) uptake was performed as previously described [62,63]. Briefly, HEK-293T cells were transfected with a combination of DAT, pcDNA3, DJ-1 and mPlum cDNA. 48 hours after transfection, cells were washed in PBS before addition of experimental media (EM), consisting of DMEM without phenol red and 1% FBS. Cells were pre-incubated with EM for 1 hour at 37°C. After the pre-incubation period, cells were imaged using an inverted fluorescence microscope. Baseline images were taken before addition of APP+. Cells were incubated with 20 nM APP+ (final concentration) and returned to the 37°C incubator for 10 min. After the 10 min incubation, cells were imaged again to measure APP+ accumulation. LUHMES cells were incubated with 2 μM APP+ (final concentrations). Images were collected under a 40X objective lens using Metamorph software (Molecular Devices, Sunnyvale, CA). APP+ images were captured with a 485/20 excitation filter and 542/27 emission filters mounted on separate filter wheels (Sutter Instruments, Novato, CA). HEK-293T cells were transfected with mPlum cDNA at 10% of the total cDNA used for the transfection. This increased the possibility that mPlum positive cells were cotransfected with DAT and either pcDNA3 or DJ-1. ROI were manually defined using mPlum fluorescence as an indicator of co-transfection. Once ROIs for all positive cells were defined, average gray values were calculated and recorded using Metamorph. Data was taken from 6 separate images that included 10 positive cells in each image. For LUHMES, images were analyzed by measuring APP+ puncta [62]. Briefly, images were thresholded with the same gray level limits for all LUHMES APP+ images and the Metamorph morphometric analysis tool was used to count the number of puncta based on area limits of 10–250 pixels.

### LUHMES cells culturing and transfection

Lund human mesencephalic (LUHMES) cell line, which is a subclone of the tetracycline-controlled, v-myc-overexpressing human mesencephalic-derived cell line MESC2.10, was obtained from ATCC (ATCC, Cat. #CRL-2927). Cells were cultured as previously described [64,65]. Briefly, LUHMES cells were cultured in tissue culture plates precoated with 10 μg/ml poly-L-ornithine and 1 μg/ml fibronectin. LUHMES cells were normally maintained in DMEM/F12 with 1x N2 supplement and 40 ng/ml bFGF. For neuronal differentiation, cells were plated at 30–50% confluency and the next day media was replaced with differentiation media (DMEM/F12, 1x N2 supplement, 1 mM dibutyryl cAMP, 1 μg/ml tetracycline, 2 ng/ml GDNF) for 2–4 days. LUHMES cells were transfected prior differentiation using Lipofectamine 2000 reagent (Life technologies) following manufacturer’s protocol. For 35 mm plates, 2 ug of cDNA (total) and 2 uL of LF2000 reagent was used. Cells were then differentiated 2 days post-transfection.

## Results

### DJ-1 regulation of DAT activity

**Fig 1:**
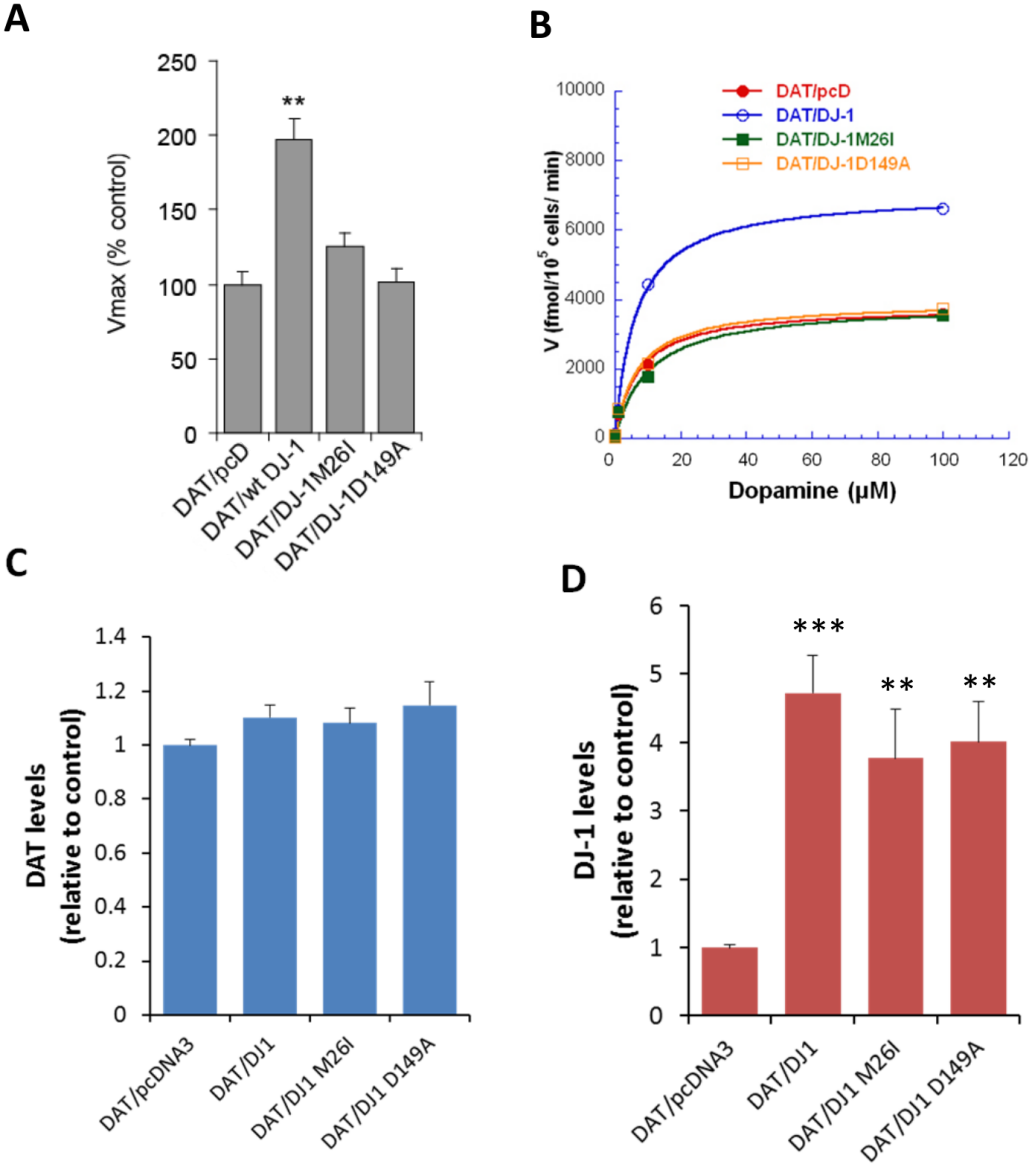
Wild-type DJ-1 up-regulates DAT activity. (A) Bar plot of [^3^H]DA uptake in HEK-293T cells co-transfected with DAT/pcDNA3 (pcD), DAT/wild-type (wt) DJ-1, DAT/M26I DJ-1, or DAT/D149A DJ-1. Cells co-expressing DAT and wt DJ-1 have significantly up-regulated DAT activity as indexed by [^3^H]DA uptake (** P<0.01, significantly different from DAT/pcD group; one-way ANOVA post hoc SNK test, n = 11). (B) Representative saturation plot of [^3^H]DA uptake in HEK-293T cells co-expressing DAT with pcDNA3, wt DJ-1, M26I DJ-1, or D149A DJ-1. Cells co-expressing DAT and wt DJ-1 exhibit an increase in V_max_ for [^3^H]DA uptake accumulation without any significant alteration in estimated affinity values (K_m_ = 3.77–8.73 μM, n = 11, one-way ANOVA, P = 0.2988). Western blots of samples that were transfected with DAT and pcDNA3, wt DJ-1, M26I DJ-1, or D149A DJ-1 were analyzed for semi-quantitative measurement of either total DAT levels (C) or DJ-1 levels (D) expressed in cells (** P<0.01, *** P<0.001 significantly different from DAT/pcDNA3 control group, n = 3).

To first determine if DJ-1 can modulate DAT function, we measured [^3^H]DA uptake activity in cells that were co-expressing DAT with either wild-type DJ-1 or the empty expression plasmid pcDNA3. As shown in Fig 1A, there is a significant increase in DAT-mediated [^3^H]DA uptake in cells co-expressing DJ-1 compared to cells co-transfected with the empty expression plasmid. Furthermore, this increase in DAT activity appears to be specific to wild-type DJ-1 as co-expression of M26I or D149A DJ-1 mutants did not enhance DAT-mediated [^3^H]DA uptake (Fig 1A and 1B). Vmax values (pmol/10^5^ cells/min) for DAT/p3, DAT/DJ-1, DAT/M26I and DAT/D149A were calculated to be 2.48 ± 0.88, 4.72 ± 1.47, 2.37 ± 0.94 and 2.65 ± 1.65, respectively. Homozygous M26I and heterozygous D149A DJ-1 mutations have been associated with early onset PD [51]. While it is unclear how exactly these mutations affect DJ-1 function, several studies suggest that the mutations can have potential effects on protein stability, aggregation dynamics and antioxidant function [66–72]. Examining the kinetics of [^3^H]DA uptake revealed that there was no significant change in the affinity for DA exhibited by the DAT upon co-expression of DJ-1 (Fig 1B). Km values (μM) of 6.29 ± 1.59, 8.73 ± 2.55, 3.77 ± 1.05 and 5.37 ± 2.53 were calculated for DAT/p3, DAT/DJ-1, DAT/M26I and DAT/D149A, respectively. In addition, the increase in [^3^H]DA uptake does not appear to be due to an increase in DAT protein levels as determined by western blots that were used to quantify the level of total DAT protein in cells co-transfected with DAT and DJ-1. As shown in Fig 1C, there was no significant change in DAT levels upon co-expression of either wild-type or mutant DJ-1 and suggests that the increase in DAT activity is due to another mechanism such as increased cell surface localization. In addition, DJ-1 expression levels were not significantly different between wildtype DJ-1 and the various mutants (Fig 1D). Western blots for both DAT and DJ-1 are shown in S1 Fig.

### Co-expression of DJ-1 leads to enhanced cell surface localization of DAT

**Fig 2:**
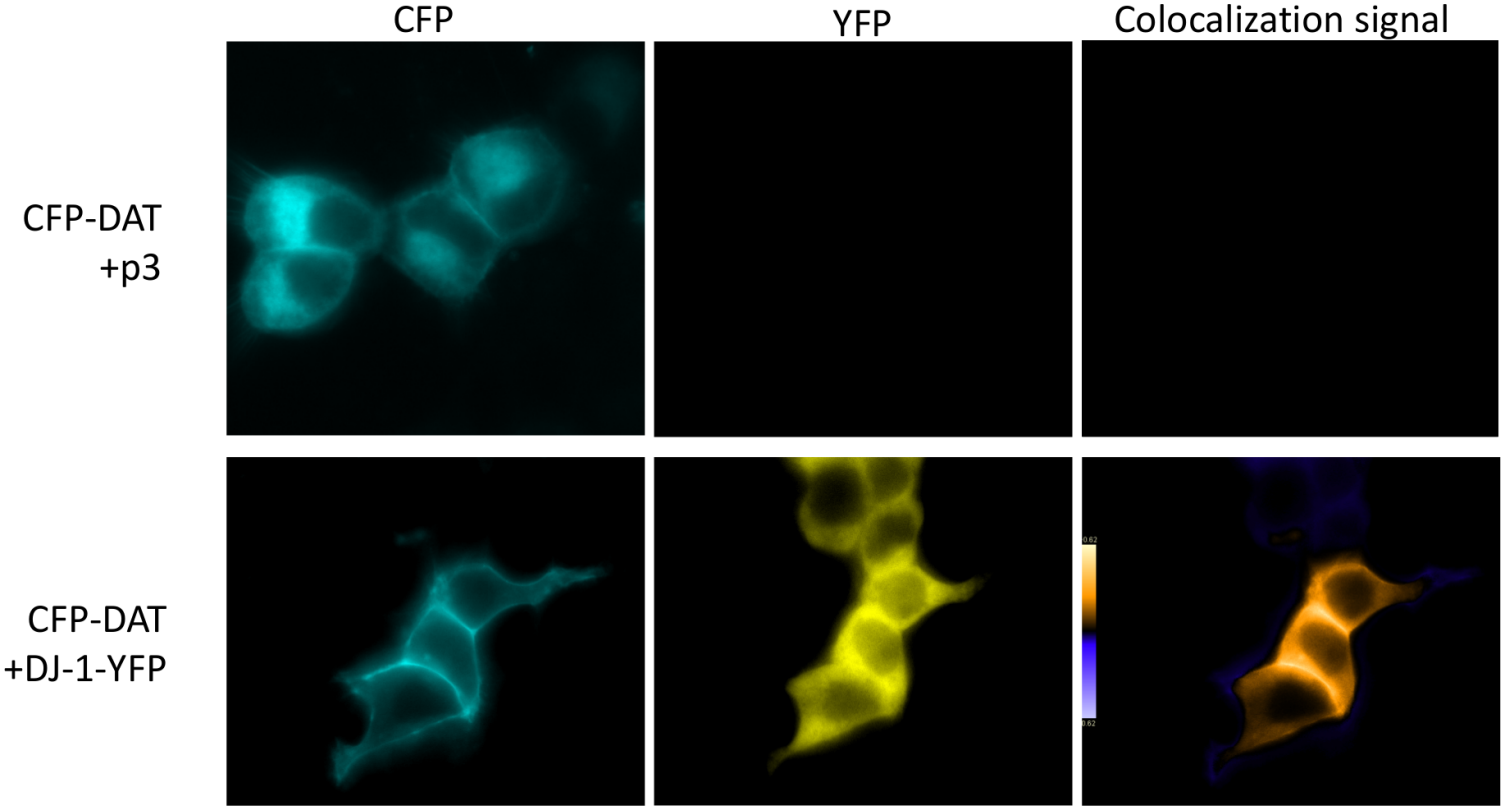
Co-expression of DJ-1 increases DAT localization at the cell surface. HEK-293T cells were transfected with CFP-DAT and either pcDNA3 or DJ-1-YFP and fluorescence live cell imaging was performed on an inverted microscope 24 hours after transfection. In cells expressing CFP-DAT alone, DAT localization occurs both intracellularly and on the cell surface. However, co-expression of DJ-1-YFP leads to a significant increase in CFP-DAT localized at the cell surface. Merged images were generated using ImageJ Intensity Correlation Analysis plugin and displays the PDM value (Product of the Differences from the Mean) for each pixel with a corresponding scale bar. Images are representative of 4 independent experiments.

To determine if the enhanced DAT mediated DA uptake upon co-expression of DJ-1 is due to increased DAT cell surface localization, we compared CFP-DAT cellular localization in cells transfected with CFP-DAT alone versus HEK-293T cells co-transfected with DJ-1-YFP. As shown in Fig 2, live imaging of cells that were transfected with CFP-DAT and the empty expression plasmid pcDNA3, 24 hours after transfection, revealed a significant population of DAT localized to intracellular compartments as well as a proportion of DAT localized at the cell surface. However, co-expression of DJ-1-YFP leads to an increase in cell surface localization of CFP-DAT, particularly in areas adjacent to other cells co-expressing both CFP-DAT and DJ-1-YFP. Although there is a significant amount of differential localization between DAT and DJ-1, co-localization of both proteins is evident throughout various regions of the cell including the cell surface. DJ-1 localization does not appear to be specific to any particular subcellular compartment but localized throughout the cell, with very low DJ-1-YFP fluorescence signal in the nucleus.

### DJ-1 forms a complex with DAT

**Fig 3:**
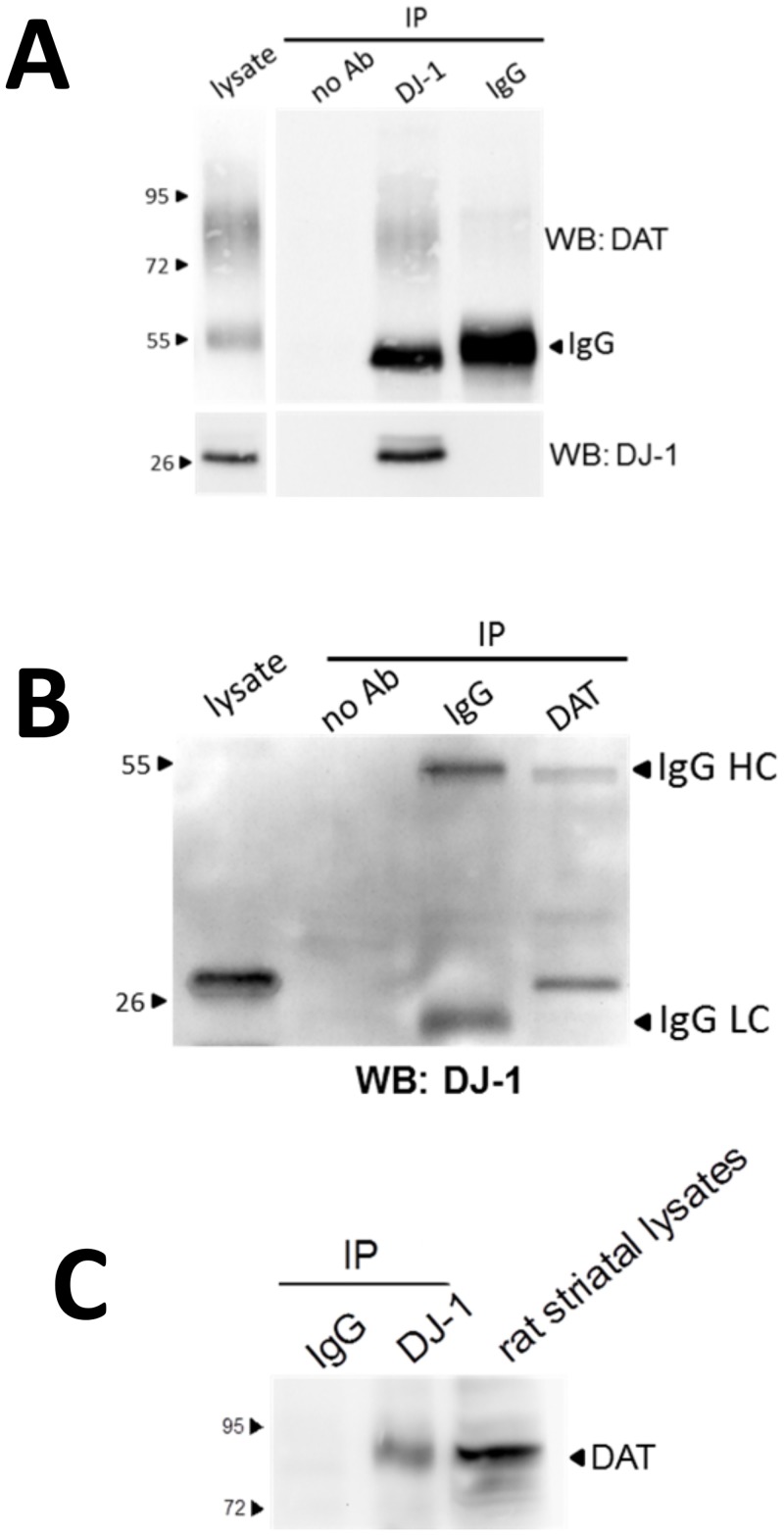
Association of DJ-1 with DAT. (A) Co-immunoprecipitation of DAT with DJ-1 was performed by incubating 500 μg of solubilized HEK-293T cells that were co-transfected with DAT and DJ-1 with 2 μg of DJ-1 antibodies or with 2 μg of mouse IgG controls. 500 ng of HEK-293T lysate was used as a positive control. The top panel shown co-immunoprecipitation of DAT with DJ-1 and the lower panel confirms immunoprecipitation of DJ-1. (B) Co-immunopreciptation of DJ-1 with DAT. 500 μg of lysates from HEK-293T co-expressing DAT and DJ-1 was incubated with 2 μg of DAT antibodies or with 2 μg of rat IgG control antibody. Five μg of HEK-293T lysate was used as a positive control. Although we use antibodies from different species for the immunoprecipitation and the western blot, there is still some crossreactivity that leads to the detection of IgG bands in the resulting immunoblots (IgG HC—heavy chain; IgG LC–light chain). (C) Co-immunoprecipitation of DAT with DJ-1 from solubilized rat striatal tissue. 750 μg of striatal tissue was immunoprecipitated with DJ-1 antibody. Resulting immunoprecipitates were run on SDS-PAGE, transferred to PVDF membranes, and blotted with DAT monoclonal antibodies.

**Fig 4:**
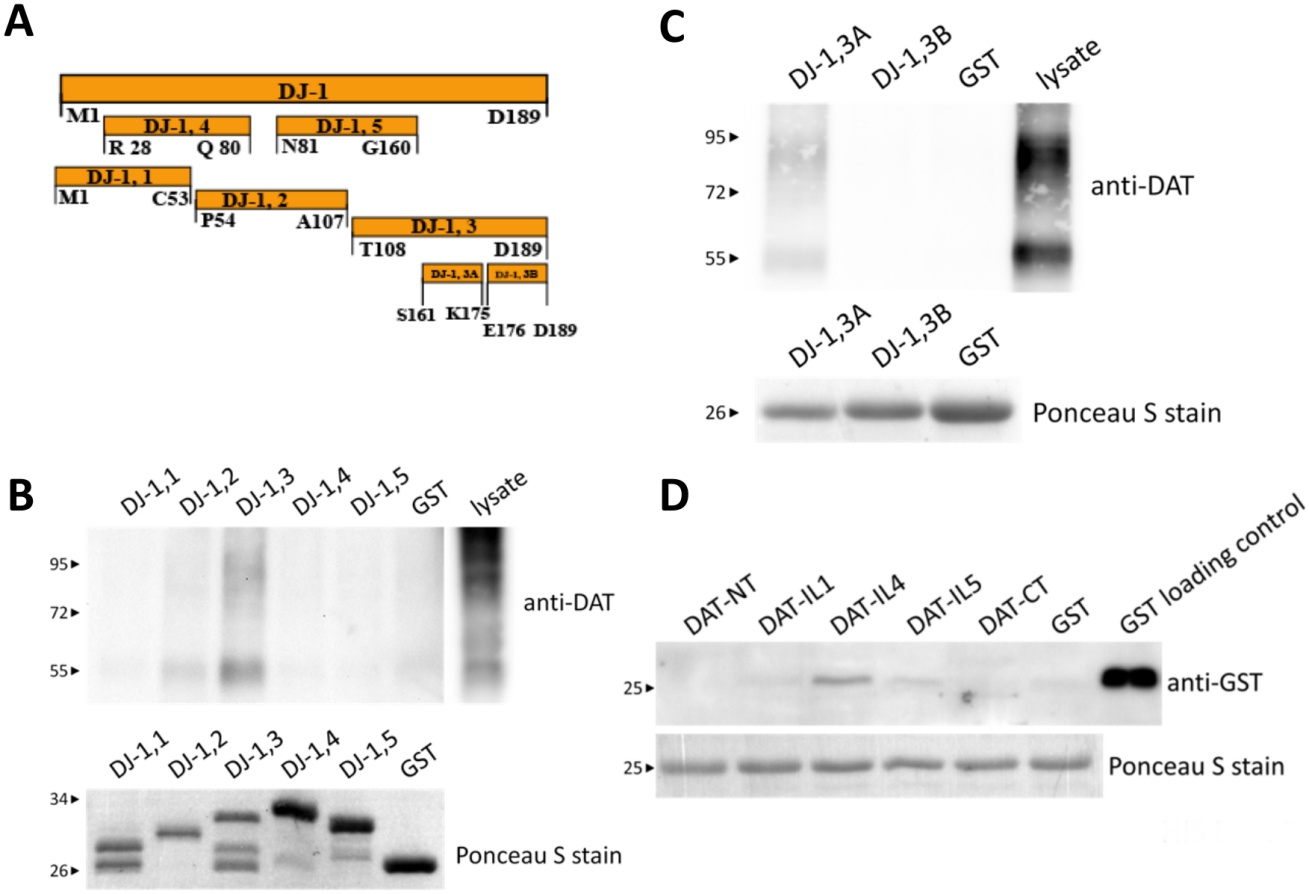
Identification of the DJ-1 domain involved in the DAT/DJ-1 interaction. (A) Schematic illustration of the different segments of DJ-1 that were used to generate GST fusion peptides and the nomenclature used for each of the various different regions. (B) Association of DJ-1,3 region with DAT. Various GST fusion peptides of DJ-1 were used to affinity purify the DAT from lysates prepared from HEK-293T cells transfected with DAT. 50 μg of HEK-293T lysate was used as a positive control. Western blots reveal the ability of the DJ-1,3 (G108-D189) region to affinity purify the DAT, while none of the other peptides were capable of pulling down DAT. Lower panel shows Ponceau S stain of blots to index the relative amounts of GST fusion peptide. GST fusion peptide levels were equivalent to or greater than the amount of GST-DJ-1,3 peptide. (C) The DJ-1,3A region mediates the interaction between DJ-1 and DAT. GST fusion peptides of DJ-1,3A and DJ-1,3B regions were used to affinity purify the DAT from lysates prepared from HEK293T cells transfected with DAT. 1 μg of HEK293T lysate was used as a positive control. Western blots show the ability of the DJ-1,3A (S161-K175) region to affinity purify DAT, while none of the other peptides were capable of pulling down DAT. Lower panel shows Ponceau S staining of blots to index the relative amounts of GST fusion peptide. GST fusion peptide levels were equivalent to or greater than the amount of GST-DJ-1,3A peptide. (D) Direct association of the intracellular loop 4 of DAT (DAT-IL4) with DJ-1. His-tagged full-length DJ-1 protein (10 μg) was used to affinity purify 0.5 μg various GST fusion peptides of DAT. GST protein alone (0.1 μg) was used as a positive control. Western blots reveal the ability of DJ-1 to affinity purify DAT-IL4, while none of the other DAT GST fusion peptides were pulled down by DJ-1. Lower panel shows Ponceau S stain of blots to index the relative amounts of His-tagged DJ-1 used in each sample.

To explore the possibility that this increase in DAT function mediated by DJ-1 is facilitated, at least in part, by a direct physical interaction we examined whether DJ-1 forms a complex with DAT through co-immunoprecipitation experiments. Immunoprecipitation of DJ-1 with anti-DJ-1 antibody separated by protein A/G-agarose beads leads to the co-precipitation of DAT in cells co-transfected with both DAT and DJ-1 (Fig 3A). Heterologous DAT expression in HEK-293T cells often leads to the detection of an immature DAT immunoreactive band at ~55 kDa and a larger diffuse band that represents the mature DAT, which is heavily glycosylated and is visualized in a Western blot as a large smear centered at ~80 kDa [33,34,73]. No bands were detected in samples that were precipitated with mouse IgG or with samples incubated with only protein A/G-agarose beads. We also show the reverse co-immunoprecipitation whereby DJ-1 also precipitates with the DAT (Fig 3B). To provide evidence that the interaction can occur in neurons we also co-immunoprecipitated DAT with DJ-1 from solubilized rat striatal lysates (Fig 3C). Next, we delineated regions within DJ-1 that are critical in the formation of the DAT/DJ-1 complex by creating GST fusion proteins that were truncations of full length DJ-1 (Fig 4A). As shown in Fig 4B, affinity purifications using GST-DJ-1,3 (T108-D189) leads to the pull-down of DAT from solubilized HEK-293T lysates. Neither the parental GST fusion protein itself nor other truncations of DJ-1 GST fusion proteins led to the purification of DAT. This suggests that the carboxyl half of DJ-1 is important in the formation of the DAT/DJ-1 complex. To define a more discrete region within the DJ-1 carboxyl terminus that is involved in the DAT/DJ-1 interaction, we created smaller truncations of the DJ1-3 region, which were named DJ-1,3A [S161-K175] and DJ-1,3B [E176-D189] (Fig 4C). Using these GST proteins in affinity purification experiments with solubilized lysates of HEK-293T expressing DAT, we were able to define a 15 amino acid segment [DJ-1,3A: S161-K175] within DJ-1 that appears to be critical for the DAT/DJ-1 complex formation.

### Direct protein-protein interaction between DAT and DJ-1

To determine if this DAT/DJ-1 complex is formed by a direct protein-protein interaction we created a purified full-length His-tagged DJ-1 protein from bacterial lysates to be used in affinity purification experiments with GST proteins that included truncated sections of the DAT. Previous studies have shown that both the amino and carboxyl terminus of the DAT are sites of interaction with various protein partners [28,36,38–42,45,47,48,74]. As shown in Fig 4D, we targeted various intracellular regions within DAT including the amino terminus (NT), intracellular loop 1 (IL1), intracellular loop 4 (IL4), intracellular loop 5 (IL5) and the carboxyl terminus (CT). When we incubated purified GST proteins with purified HIS-tagged DJ-1 only DAT-IL4 showed significant purification with His-tagged DJ-1 as demonstrated in Fig 4D. Therefore, this data provides two critical pieces of information: (i) the DAT/DJ-1 complex is potentially formed by a direct protein-protein interaction and (ii) the region within DAT that is critical for this interaction lies within DAT intracellular loop 4.

### Disruption of the DAT/DJ-1 complex with mini-genes

**Fig 5:**
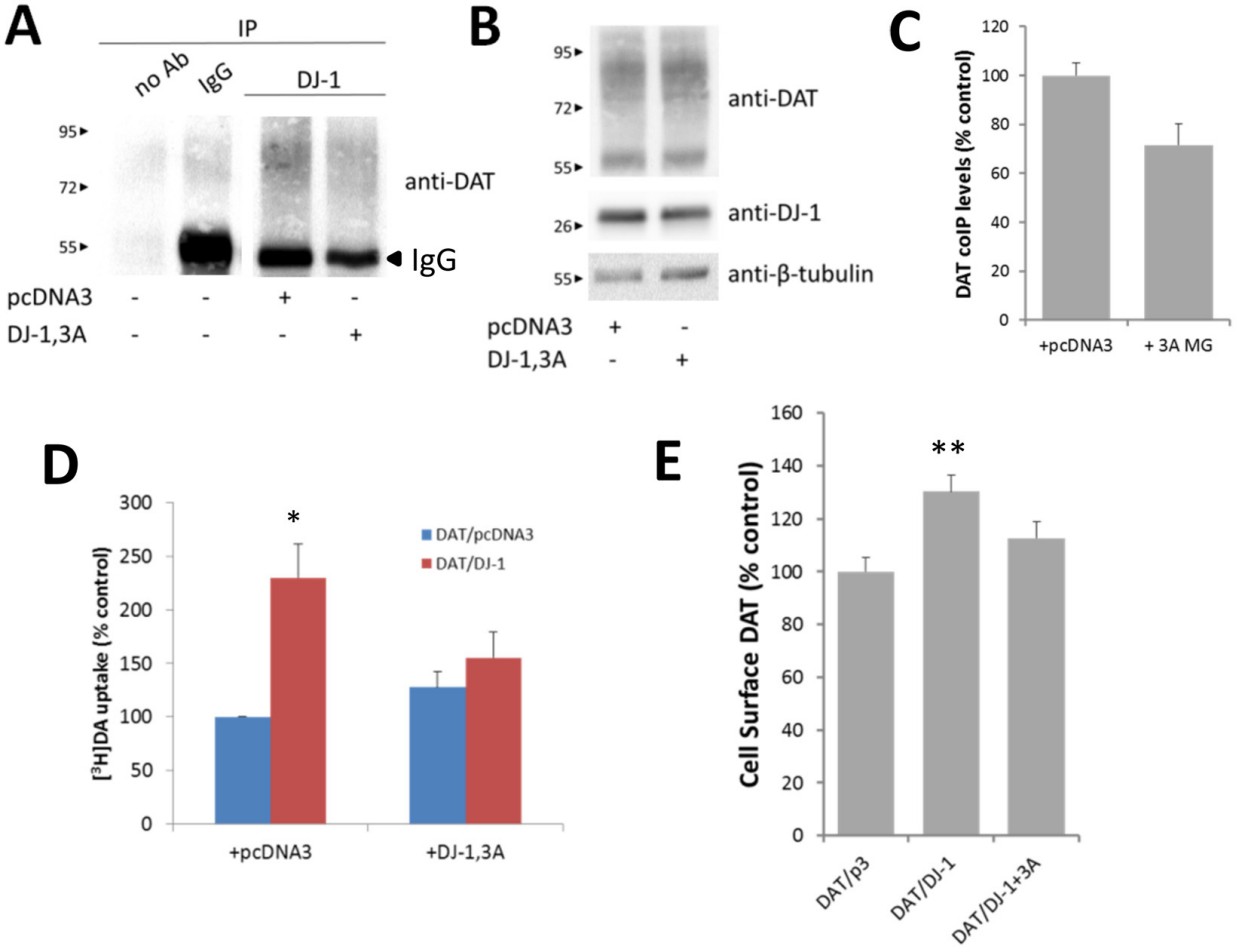
Mini-gene DJ-1,3A (DJ-1 CT161-175) blocks the interaction between DJ-1 and DAT. (A) Co-expression of the DJ-1,3A mini-gene blocks the physical interaction between DJ-1 and DAT. Co-immunoprecipitation of DAT with DJ-1 was performed by incubating 500 μg of solubilized HEK-293T cells that were co-transfected with DAT, DJ-1, and either pcDNA3 or DJ-1,3A. Western blot reveals that the DAT/DJ-1 interaction is blocked by the addition of DJ-1,3A mini-gene. (B) 2.5 μg of HEK-293T lysates were run on SDS-PAGE and immunoblotted with DAT, DJ-1 or β-tubulin. (C) Quantification of co-immunoprecipitation of DAT with DJ-1 in HEK-293T cells co-transfected with pcDNA3 or the DJ-1,3A mini-gene* P<0.05, t-test. n = 7. (D) Mini-gene DJ-1,3A blocks the functional interaction between DJ-1 and DAT. [^3^H] DA uptake was measured in HEK-293T cells that were co-expressing the DJ-1,3A mini-gene. DJ-1-mediated increase in DAT-mediated [^3^H] DA uptake was blocked with the co-expression of mini-gene encoding DJ-1,3A but does not alter the uptake in cells only transfected with DAT. (* P<0.05, significantly different from DAT/pcD group; # P<0.05, significantly different from DAT/DJ-1 group; one-way ANOVA post hoc Tukey test, n = 3). (E) Quantification of DAT cell surface localization using a cell-based ELISA. HEK-293T cells co-expressing DAT and DJ-1 exhibit an approximate 30% increase in DAT cell surface localization compared with cells co-transfected with DAT and pcDNA3, an effect blocked by the co-expression of the DJ-1,3A mini-gene (** P<0.01 compared to DAT/p3 group; one-way ANOVA, post hoc Tukey test, n = 6).

To examine the effects of disrupting the physical interaction between DAT/DJ-1, we co-transfected mini-genes that encode the sequence within DJ-1,3A [S161-K175] that would compete with wild-type DJ-1 for binding to the DAT. As shown in Fig 5A, when indexed through co-immunoprecipitation assays there is a significant disruption in the DAT/DJ-1 interaction in cells that are co-expressing the DJ-1,3A mini-gene compared to cells co-transfected with the empty expression plasmid. To verify that the difference in co-immunoprecipitation is not due to changes in DAT or DJ-1 expression levels induced non-specifically by co-transfection of the DJ-1,3A mini-gene, we measured the levels of both DAT and DJ-1 in our samples. As seen in Fig 5B, there was no significant difference in expression levels of either DAT or DJ-1 with DJ-1,3A mini-gene co-transfection. Quantification of DAT/DJ-1 co-immunoprecipitation results reveals an approximate 30% decrease in DAT/DJ-1 co-immunoprecipitation levels as a result of DJ-1,3A mini-gene co-transfection (Fig 5C). As observed previously, cells co-transfected with DAT and DJ-1 exhibited a significant increase in [^3^H]DA uptake compared to cells transfected with DAT alone. This increase in DA uptake was blocked in cells that were co-transfected with the DJ-1,3A mini-gene. Moreover, this effect appears to be specific to the DAT/DJ-1 interaction as the DJ-1,3A mini-gene had no effect on cells expressing DAT alone (Fig 5D). Therefore, the data appears to confirm that the DAT/DJ-1 direct interaction facilitates the increase in DAT activity. We have previously shown an increase in DAT cell surface localization with co-expression of DJ-1 (Fig 2). To quantify these results and to examine the effects of the DJ-1,3A mini-gene we examined DAT cell surface levels in HEK-293T cells co-transfected with DAT and either pcDNA3, DJ-1 or DAT and the DJ-1,3A mini-gene using a cell based ELISA assay. We measured cell surface localization using an antibody that recognizes an epitope on the 2^nd^ extracellular loop of the DAT. As shown in Fig 5E, there is a 30% increase in DAT cell surface localization with co-expression with DJ-1. This increase in plasmalemmal DAT is blocked with coexpression of the DJ-1,3A mini-gene.

### Knockdown of endogenous DJ-1 leads to decreases in DAT activity

**Fig 6:**
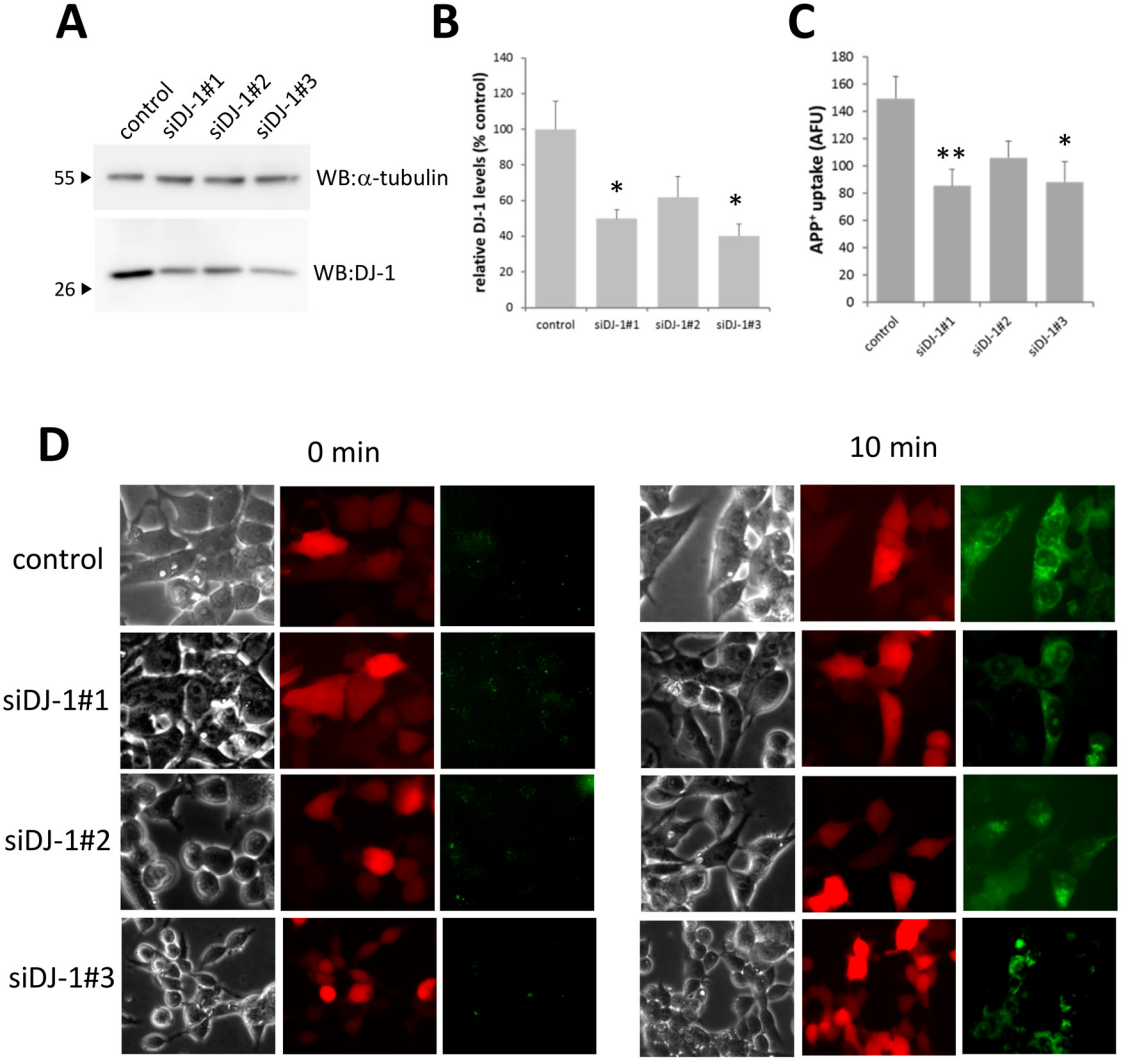
Knockdown of endogenous DJ-1 leads to decreased DA reuptake. (A) Western blots for DJ-1 taken from cells that were transfected with either control or 3 different siRNA molecules (siDJ-1#1, siDJ-1#2, siDJ-1#3; Integrated DNA Technologies) were used to induce knockdown of endogenous DJ-1 expressed in HEK-293T cells. Representative immunoblots are shown. (B) The levels of DJ-1 expression was quantified to show significant knockdown of DJ-1 levels in cells transfected with siDJ-1#1 and siDJ-1#3 (50–60% decrease compared to controls), while siDJ-1#2 exhibited a decreasing trend in DJ-1 expression. * P<0.05 vs control group; one-way ANOVA post hoc Tukey test, n = 3. (C and D) DAT function was measure by measuring APP+ uptake levels. Cells were incubated with 20 nM APP+ for 10 min at 37°C, upon which they were imaged with an inverted fluorescent microscope to measure APP+ accumulation. Experiments revealed a significant decrease in APP+ uptake in cells transfected with siDJ-1#1 and siDJ-1#3, while there was decreasing trend in APP+ uptake in cells transfected with siDJ-1#2. * P<0.05, ** P< 0.01 vs control group; one-way ANOVA post hoc Tukey test, n = 3 (8–9 cells from each group was measured in each experiment, for a total of 26 cells per group).

To further validate the effects of DJ-1 on DAT activity we depleted the levels of endogenous DJ-1 in HEK-293T cells using siRNA-mediated gene knock-down. Using 3 different DJ-1 specific siRNAs, we show significant knockdown in DJ-1 transcript with all DJ-1 siRNAs (S2 Fig) but only 2 of the 3 DJ-1 siRNAs (siDJ-1#1, siDJ-1#3) exhibited significant reduction in DJ-1 protein levels compared to cells transfected with NC-1 control siRNA (Fig 6A and 6B). The other DJ-1 specific siRNA (siDJ-1#2) exhibited lower DJ-1 protein levels but was not statistically different from control (Fig 6B). We then assessed DAT activity in these cells by measuring APP+ accumulation. APP+ is an analogue of MPP+, a neurotoxin and known substrate for the DAT. APP+ has been previously used as a fluorescent substrate to index DAT activity [62,63]. Incubating cells with 20 nM APP+ for 10 min led to significant APP+ accumulation (Fig 6C and 6D) that was significantly reduced in cells transfected with siDJ-1#1 and siDJ-1#3. Cells transfected with siDJ-1#2 exhibited reduced APP+ accumulation but was not statistically different from the control group. We observed similar reductions in [^3^H]DA uptake in co-transfected HEK-293T cells (S2 Fig).

### DAT-mediated uptake in LUHMES neuronal cells

**Fig 7:**
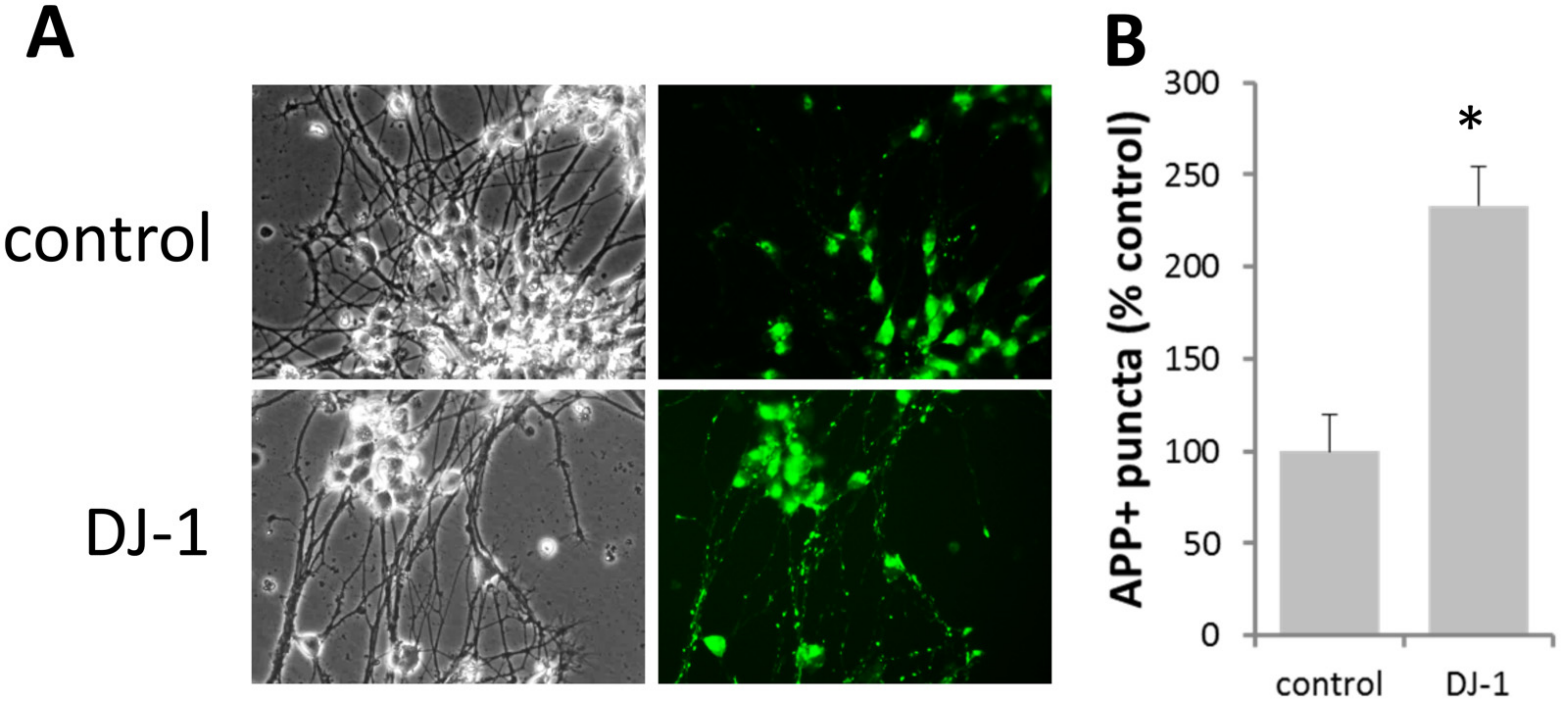
APP+ uptake in differentiated LUHMES neurons is significantly affected by DJ-1 expression. (A) Differentiated LUHMES neurons that were previously transfected with DAT cDNA and with either pcDNA3 (control) or DJ-1 were incubated with 10 μM APP+ for 30 min. Cells were subsequently imaged and analyzed for APP+ puncta as an index of DAT activity. Puncta was defined by thresholding images at defined gray levels and with specific spatial parameters that excluded objects larger than 250 pixels^2^ and smaller than 10 pixels^2^. (B) Indexing puncta levels between cells transfected with DAT/pcDNA3 and DAT/DJ-1 showed a significant increase in APP+ puncta upon co-expression of DJ-1 (* P<0.05, t-test, n = 3). For quantification purposes we excluded APP+ accumulation in cell bodies.

To examine the effects of the DAT/DJ-1 interaction in a more relevant milieu we transfected immortalized Lund human mesencephalic (LUHMES) cells with pcDNA3 or DJ-1 and measured APP+ accumulation. Although LUHMES cells have been shown to express DAT when differentiated into post-mitotic neurons [65] we also co-transfected with DAT cDNA to allow for significant APP+ accumulation. APP+ assays have previously been used to measure DAT activity in acute rat midbrain and striatal slices [62]. Karpowicz et al have identified that APP+ accumulation was distinctly punctate and was significantly reduced when pretreated with the DAT inhibitor nomefinsine [62]. Therefore, we also quantified APP+ puncta in our LUHMES neuronal cells as an index of DAT mediated APP+ accumulation. In these neuronal cells treatment with 10 μM APP+ for 30 min led to significant increase in puncta with neurons transfected with DJ-1 compared to control neurons (Fig 7). There was no significant difference in APP+ accumulation in the cell bodies of either control or DJ-1 expressing LUHMES neuronal cells (data not shown).

## Discussion

While the physiological function of DJ-1 remains unclear several studies have shown DJ-1 to be involved in a wide variety of cellular functions ranging from transcriptional regulation [60,75–77], regulating kinase activities [78–81], serving as a protein chaperone or protease [82–84] and an anti-oxidant factor [77,85,86]. Furthermore, studies have shown that DJ-1 can interact with a wide variety of proteins including apoptosis signal-regulating kinase 1 [87], Hsp70 [88], α-synuclein [84,89–91], Daxx [78], MEKK1 [92], DJBP [76], HIPK1 [79], Topors/p53BP3 [93], PIASxα [94] and TTRAP [95]. In addition, there is evidence that DJ-1 plays a role in dopaminergic neurotransmission. DJ-1 is expressed in the terminals of DA neurons [96] and DJ-1 null mice appear to exhibit increased DA reuptake [54,56] while exhibiting decreased D2 autoreceptor function [54,57], two key elements in regulating DA levels, which corresponds to increased DA tissue content in mice lacking DJ-1. Furthermore, DJ-1 null mutant mice exhibit enhanced sensitivity to MPTP resulting in increased striatal denervation by DA neurons [55,56]. This increased susceptibility to MPTP in DJ-1 null mice was ameliorated through viral-mediated expression of DJ-1 [55]. Furthermore, DJ-1 knockdown in *Drosophila* exhibited cellular accumulation of ROS, hypersensitivity to oxidative stress, dysfunction and degeneration of DA and photoreceptor neurons [81]. Similarly, DJ-1 knockdown in zebrafish also exhibit a loss of DA neurons after treatment with H_2_O_2_ and the proteasome inhibitor MG132 [97]. DJ-1 has also been shown to upregulate tyrosine hydroxylase (TH) activity [98–100]. Taken together, the knockout and knockdown studies have uncovered a potential role for DJ-1 in regulating DA activity and potentially mediating protective responses towards oxidative stress. Our study provides additional evidence that DJ-1 may play a role in dopamine neurotransmission. Here we have shown that DJ-1 can interact directly with the DAT and appears to functionally upregulate DAT activity as indexed by enhanced DA uptake, which is surprising considering DJ-1 knockout mice appear to exhibit increased DAT activity. We speculate that this may provide a mechanism that allows DJ-1 to differentially modify DAT activity in the presence of oxidative stress or elevated DA levels, which is buttressed by the observation that dopamine quinones can covalently bind to DJ-1 at residue Cys-106 and induce destabilization through conformational changes in several domains within DJ-1 including residues 155–187, which encompasses the region that interacts with the DAT [101]. Another potential explanation may be that DJ-1 can modulate DAT-mediated DA efflux. However, amphetamine or 17β-estradiol induced DAT–mediated efflux does not appear to be affected by DJ-1 co-expression in HEK-293T cells (S3 Fig). While we are uncertain of the cause for the discrepancy between our data and the DJ-1 knockout studies in mice that show a potential increase in DA reuptake, we speculate that this may be attributed to another function of DJ-1, transcriptional regulation. Previous studies have shown that either inactivation of DJ-1 or overexpression of DJ-1 leads to changes in TH expression [98–100]. Other studies suggest that DJ-1 is involved in the transcriptional regulation of cholecystokinin [102], low-density lipoprotein receptor [103], thioredoxin 1 [104], Bax [105]. In addition, siRNA mediated knockdown of DJ-1 can lead to increased transcription of several genes suggesting that DJ-1 may also exhibit repressor function for certain genes [106]. Therefore, if DJ-1 exhibits a similar repressor function for the DAT gene, then it would not be surprising to discover that DJ-1 knockout mice exhibit increased DAT activity. Furthermore, in our experiments, DJ-1 repressor activity would not be asserted on heterologous expression of DAT cDNA. Another factor that may explain the discrepancy of our data with the DJ-1 knockout studies is that some DJ-1 effects appear to be species specific. Ishikawa *et al*. suggest that the PSF/DJ-1 complex that impacts TH transcription only occurs with the human promoter and not the mouse TH promoter [99]. Nevertheless, it is conceivable that DJ-1 may impact DAT function both transcriptionally and through a direct protein-protein interaction. However, we do not believe that the effects we have observed are due to transcriptional regulation of the transporter by DJ-1 since there are no changes in total DAT levels as a consequence of the DJ-1 co-expression. Furthermore, disrupting the physical interaction between DJ-1 and DAT also inhibits the functional upregulation of DAT activity.

The DAT belongs to a family of Na^+^/Cl^-^ dependent catecholamine transporters. The regulation of the transporter is important as it is the primary mechanism by which DA is removed from the extracellular space after diffusing out of the synapse. In recent years several proteins have been identified to directly interact with the DAT and modulate its function [28,36,38–48]. Several other proteins have also been shown to modify DAT function including the D3 receptor [107], Kappa opioid receptor [108], MAPK [109] and Parkin [110] but it is unclear if these interactions are facilitated by a direct protein-protein interaction. Dopamine itself can potentially generate reactive oxygen species (ROS) through both enzymatic and non-enzymatic oxidation reactions. Accumulation of these oxidative metabolites can lead to oxidative stress, which can damage proteins, lipids and DNA [111–113]. Therefore, a dysregulation of cytoplasmic DA levels could potentially generate pathological oxidative stress and may serve as a common pathway in both sporadic and familial forms of PD leading to nigral degeneration. Transgenic mice that either overexpress DAT [114] or exhibit low expression of VMAT2 [115], which would contribute to increased cytosolic DA levels, exhibit neurodegeneration in affected neurons. Thus, it is conceivable that altered DAT activity could facilitate pathological intracellular accumulation of DA or DA-like molecules, which would subsequently lead to the production of reactive free radical metabolites that are neurotoxic to dopaminergic cells. Moreover, disease-causing mutations in DJ-1 may lead to disruption of the normal mechanisms that regulate DAT function and contribute to accumulation of ROS that participate in the pathophysiology that leads to dopaminergic cell death. Our study suggests that further investigations in the role of DJ-1 in dopaminergic function appear to be warranted. In addition, the direct interaction between DAT and DJ-1 adds to the complexity of proteins that regulate DAT activity. The landscape of proteins that interact directly with the DAT provides a rich mosaic by which transporter function is modified and it will be important to understand the spatial and temporal intricacies of these interactions.

## Supporting Information

S1 Fig DAT and DJ-1 expression levels in HEK-293T cells. Western blots using lysates prepared from cells co-transfected with DAT and either pcDNA3, wildtype (wt) DJ-1, M26I DJ-1 mutant or D149A DJ-1 mutant. Each lane was loaded with 5 μg of soluble lysates. The top panel shows DAT protein levels while the middle panel shows DJ-1 levels, while the bottom panel reveals α-tubulin levels, which is used as loading control. Quantification of these blots are shown in Fig 1C and 1D.
(PDF)

S2 Fig siRNA mediated knockdown of DJ-1. HEK-293T cells transfected with NC-1 control siRNA or DJ-1 specific siRNA were examined for DJ-1 transcript levels through real time PCR. (A) HEK-293T cells transfected with DJ-1 specific siRNA siDJ-1#1, siDJ-1#2 or siDJ-1#3 exhibited a significant decrease in DJ-1 transcript levels (*** P<0.001, one way ANOVA post hoc Tukey test, n = 3). (B) Cells transfected with DAT and DJ-1 specific siRNA siDJ-1#1 show a significant decrease in [^3^H]DA uptake compared to controls. While not statistically different, siDJ-1#2 and siDJ-1#3 transfected cells also exhibited reduced [^3^H]DA uptake levels (* P <0.05, one way ANOVA post hoc Tukey test, n = 6).
(PDF)

S3 Fig DAT-mediated [^3^H]DA efflux. (A) DAT-mediated [^3^H]DA efflux induced by treating HEK-293T cells with 1 nM 17β-estradiol for 10 min. Cells were transfected with DAT and pcDNA3, DJ-1, DJ-1,3A mini-gene (3A) or both DJ-1 and the 3A mini-gene. Both levels of extracellular and intracellular levels of [^3^H]DA was measured and the ratio of extracellular/intracellular was used as an index of DA release. No statistical differences were observed (n = 4) (B) [^3^H]DA release was measured in HEK-293T cells treated with 10 μM amphetamine for 20 min. Cells were co-transfected with DAT cDNA and siRNA duplexes (NC-1 control, siDJ-1#1, siDJ-1#2 or siDJ-1#3). DA release was quantified by the ratio of extracellular/intracellular [^3^H]DA levels. No statistical differences were observed (n = 3).
(PDF)

S1 Methods Supplemental Methods. (PDF)
