# Supplementary figures and images for: A Physical Interaction between the Dopamine Transporter and DJ-1 Facilitates Increased Dopamine Reuptake
Source: PLoS One. 2015 Aug 25;10(8):e0136641. doi: 10.1371/journal.pone.0136641 (PMC4549284; doi:10.1371/journal.pone.0136641)

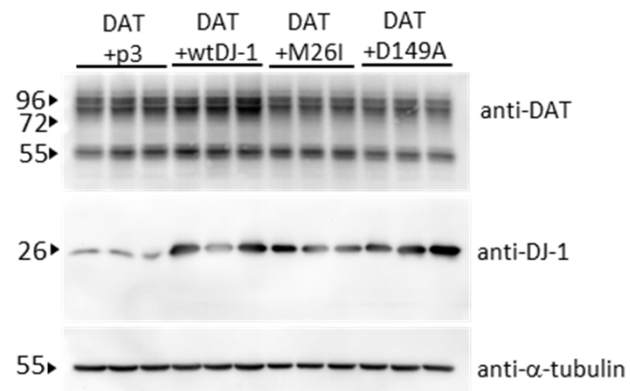

Figure S1

Supplement: S1 Fig — Western blots using lysates prepared from cells co-transfected with DAT and either pcDNA3, wildtype (wt) DJ-1, M26I DJ-1 mutant or D149A DJ-1 mutant. Each lane was loaded with 5 μg of soluble lysates. The top panel shows DAT protein levels while the middle panel shows DJ-1 levels, while the bottom panel reveals α-tubulin levels, which is used as loading control. Quantification of these blots are shown in Fig 1C and 1D. (PDF) [file pone.0136641.s001.pdf]

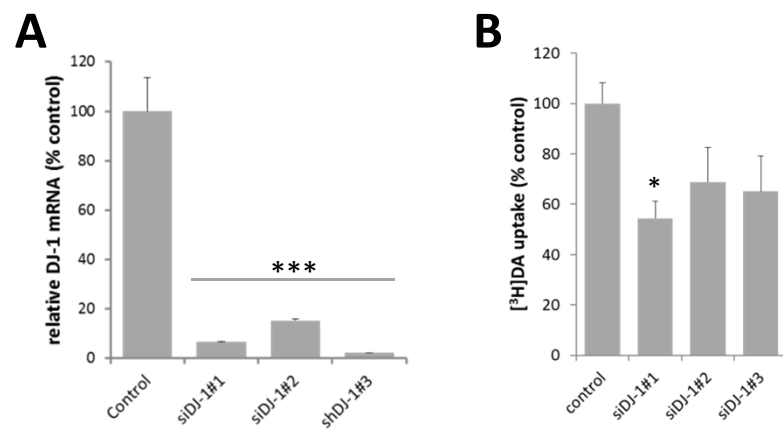

Figure S2

Supplement: S2 Fig — HEK-293T cells transfected with NC-1 control siRNA or DJ-1 specific siRNA were examined for DJ-1 transcript levels through real time PCR. (A) HEK-293T cells transfected with DJ-1 specific siRNA siDJ-1#1, siDJ-1#2 or siDJ-1#3 exhibited a significant decrease in DJ-1 transcript levels (*** P<0.001, one way ANOVA post hoc Tukey test, n = 3). (B) Cells transfected with DAT and DJ-1 specific siRNA siDJ-1#1 show a significant decrease in [3H]DA uptake compared to controls. While not statistically different, siDJ-1#2 and siDJ-1#3 transfected cells also exhibited reduced [3H]DA uptake levels (* P <0.05, one way ANOVA post hoc Tukey test, n = 6). (PDF) [file pone.0136641.s002.pdf]

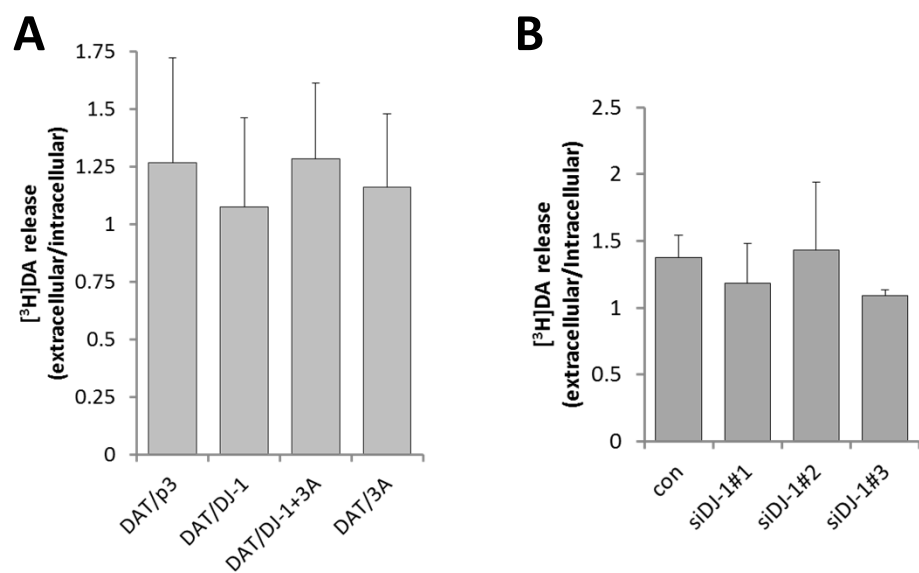

Figure S3

Supplement: S3 Fig — (A) DAT-mediated [3H]DA efflux induced by treating HEK-293T cells with 1 nM 17β-estradiol for 10 min. Cells were transfected with DAT and pcDNA3, DJ-1, DJ-1,3A mini-gene (3A) or both DJ-1 and the 3A mini-gene. Both levels of extracellular and intracellular levels of [3H]DA was measured and the ratio of extracellular/intracellular was used as an index of DA release. No statistical differences were observed (n = 4) (B) [3H]DA release was measured in HEK-293T cells treated with 10 μM amphetamine for 20 min. Cells were co-transfected with DAT cDNA and siRNA duplexes (NC-1 control, siDJ-1#1, siDJ-1#2 or siDJ-1#3). DA release was quantified by the ratio of extracellular/intracellular [3H]DA levels. No statistical differences were observed (n = 3). (PDF) [file pone.0136641.s003.pdf]
