# Supplementary material for: A Physical Interaction between the Dopamine Transporter and DJ-1 Facilitates Increased Dopamine Reuptake
Source: PLoS One. 2015 Aug 25;10(8):e0136641. doi: 10.1371/journal.pone.0136641 (PMC4549284; doi:10.1371/journal.pone.0136641)
Supplement: S1 Methods — (PDF) [file pone.0136641.s004.pdf]

S1 Supplemental Methods

cDNA synthesis and real-time PCR

HEK-293T cells transfected with NC1 control or DJ-1 specific siRNAs were collected 48 hours after transfection and total RNA was extracted with TRI reagent (Sigma, Cat.#T9424) according to the manufacturer's protocol. Approximately, 0.25 - 0.5 µg of RNA was reverse-transcribed using Superscript II (Life Technologies, Cat. # 18064-014) according to the manufacturer's protocol using an anchored oligo $(dT)_{20}$ primer. The cDNA was quantified by real time PCR using SYBR Advantage qPCR Premix (Clontech, Cat. # 638320), Rox as an internal standard and a StepOne Real Time PCR System (Life Technologies). A relative standard curve in which the sample was diluted resulting in dilutions of 1:2, 1:10, 1:50, 1:250 and 1:1,250 whereas the samples were originally diluted 1:15. Primers used for the RT-PCR experiments were; DJ-1 (5'– CTTAGAGAAACAGGCCGTTAGG – 3' and 5' – GTAGTAAGGACAGCGACTTCTG – 3'), actin (5' – TCAGAAAGATTCCTACGTGGGCGA – 3' and 5' – TGTGGTGCCAGATCTTCTCCATGT – 3').

$[^3H]$DA efflux assay

Efflux assays were performed as described previously [1,2]. Briefly, for 17β-estradiol (Sigma, Cat.# E8875) experiments cells were preincubated in uptake buffer (25 mM HEPES, 120 mM NaCl, 5 mM KCl, 1.2 mM MgSO4, 2.5 mM $CaCl_2$, 2 mg/ml glucose, 0.2 mg/ml ascorbic acid, 1 µM pargyline, 1 mM tropolone, pH 7.4) containing 50 nM desipramine (Sigma, Cat. #D3900) for 60 min at 37°C. Nonspecific efflux was measured in wells pretreated with 100 nM GBR12909 (Sigma, Cat. # D052). After the 1 hour preincubation, wells were washed in uptake buffer then 20 nM of $[^3H]$DA was added to the well for 10 min at room temperature. cells were washed in release

buffer (25 mM HEPES, 120 mM NaCl, 5 mM KCl, 1.2 mM MgSO4,2 mg/ml glucose, 0.2 mg/ml ascorbic acid, pH 7.4) then incubated with 1 nM 17β-estradiol in release buffer for 10 min at 37°C. Nonspecific release was indexed by co-incubating with 1 µM GBR12909. Release buffer was collected in scintillation vials. Cells were subsequently washed before adding 1% SDS and collecting lysates into scintillation vials. Radioactivity was measured using a Beckman liquid scintillation counter (LS 6000SC). For amphetamine-induced release, experiments were performed similarly. Cells were preincubated in uptake buffer for only 15 min with nonspecific wells also treated with 50 nM desipramine and 100 nM GBR12909. For [^{3}H]DA loading, cells were incubated in 20 nM [^{3}H]DA for 60 min at 37°C and subsequently cells were incubated with 10 µM amphetamine for 20 min at 37°C. Samples were collected in scintillation vials and measured in a Beckman liquid scintillation counter (LS 6000SC). Data was expressed as ratio for radioactivity measure in the extracellular/intracellular samples.

Supplemental References

1. Eshleman AJ, Henningsen RA, Neve KA, Janowsky A. Release of dopamine via the human transporter. Mol Pharmacol. 1994;45: 312–316. Available: http://www.ncbi.nlm.nih.gov/entrez/query.fcgi?cmd=Retrieve&db=PubMed&dopt=Citation&list_uids=7906856

2. Alyea RA, Watson CS. Nongenomic mechanisms of physiological estrogen-mediated dopamine efflux. BMC Neurosci. 2009;10: 59. doi:10.1186/1471-2202-10-59
